# Supplementary material for: Conformational Variation in Enzyme Catalysis: A Structural Study on Catalytic Residues
Source: J Mol Biol. 2022 Apr 15;434(7):167517. doi: 10.1016/j.jmb.2022.167517 (PMC9005782; doi:10.1016/j.jmb.2022.167517)
Supplement: Supplementary data 1 [file mmc1.docx]

# Variation and flexibility in enzyme catalysis: A structural study on catalytic residues

Ioannis G. Riziotis, António J. M. Ribeiro, Janet M. Thornton

European Bioinformatics Institute (EMBL-EBI), Wellcome Genome Campus, Cambridge, UK

# Supplementary Material

## Active sites dynamic superposition algorithm

For two homologous active sites *p* and *q*:

Functional atoms 3D coordinate sets *P* and *Q* respectively, of equal dimensions $N\times3$, are extracted, where *N* is the number of atoms. Coordinates *Q* are initially superposed to *P*, applying the Kabsch algorithm in a single step, followed by calculation of unweighted *RMSD*

$$RMSD= \frac{1}{N}\sqrt{\sum_{i=1}^{N} d_{i}^{2}}$$

where *d_i_* is the pairwise Euclidean distance of two corresponding atoms. This initial RMSD value is used to compute a scaling factor *c* used downstream in the calculation of atom weights. The scaling factor is calculated by a sigmoid function, defined empirically, after examining the superposition of active sites within several homologous enzyme families

$$c=\frac{9}{1+e^{\left( -1.7RMSD-5.3 \right)}}+1$$

Atom weights (as vector *W*) are derived by incorporating the pairwise atom Euclidean distances into a Gaussian function, scaled by the scaling factor from the previous step, as

$$W= \left( \begin{matrix} w_{i=1}, & \cdots& ,w_{i=N} \end{matrix} \right)$$

where

$$w_{i}=e^{-\frac{d_{i}^{2}}{c}}$$

Next, the centroids of the two coordinate sets are calculated as

$C_{P}=\left( \begin{matrix} \frac{1}{N}\sum_{i=1}^{N} w_{i}x_{Pi} \\ \frac{1}{N}\sum_{i=1}^{N} w_{i}y_{Pi} \\ \frac{1}{N}\sum_{i=1}^{N} w_{i}z_{Pi} \end{matrix} \right)$ , $C_{Q}=\left( \begin{matrix} \frac{1}{N}\sum_{i=1}^{N} w_{i}x_{Qi} \\ \frac{1}{N}\sum_{i=1}^{N} w_{i}y_{Qi} \\ \frac{1}{N}\sum_{i=1}^{N} w_{i}z_{Qi} \end{matrix} \right)$

Coordinates are then translated to the origin of the cartesian space by subtracting the weighted centroids

$$P^{'}=P-C_{P}$$

$$Q'=Q-C_{Q}$$

The next step is the calculation of a weighted cross-covariance matrix between the two coordinate sets

$$K_{P'Q'}={(P'}^{T}W)Q'$$

The cross-covariance matrix is factorised through singular value decomposition (SVD)

$$K_{P^{'}Q^{'}}=U\Sigma V^{T}$$

Rotation matrix is calculated as

$$R={(VU^{T})}^{T}$$

Translation vector is calculated as

$$T=C_{Q}-C_{P}R$$

*C_Q_* coordinates are superposed on the *C_P_* coordinates by applying a transformation operation using the rotation matrix *R* and translation vector *T*

$$Q^{''}= Q^{'}R+T$$

Finally, the same atom weights are incorporated into *RMSD* calculation (weighted *RMSD*, or *wRMSD*)

$$wRMSD= \frac{1}{N}\sqrt{\sum_{i=1}^{N} w_{i}d_{i}^{2}}$$

The process is repeated by calculating new weights, weighted transformation and *wRMSD* until a *wRMSD* convergence of < 0.0001Å is achieved, with a maximum of 6000 iterations. In a few cases where convergence cannot be achieved, unweighted superposition is performed in 10 cycles, applying simple rejection of outlying atoms, with a pairwise distance threshold of 5Å. After successful superposition of the homologous active sites, a final, unweighted *RMSD* value over all functional atom triads is calculated. This approach not only accomplishes optimal, flexibility-aware superposition, but also outputs an *RMSD* value proportional to the overall structural dissimilarity of the active sites. The reason why unweighted *RMSD* is more appropriate for our purposes is because *wRMSD,* in principle, reflects the dissimilarity of only the most rigid parts of a structure, weighting down the more variable parts, therefore it is not informative of the overall dissimilarity.

## Figures


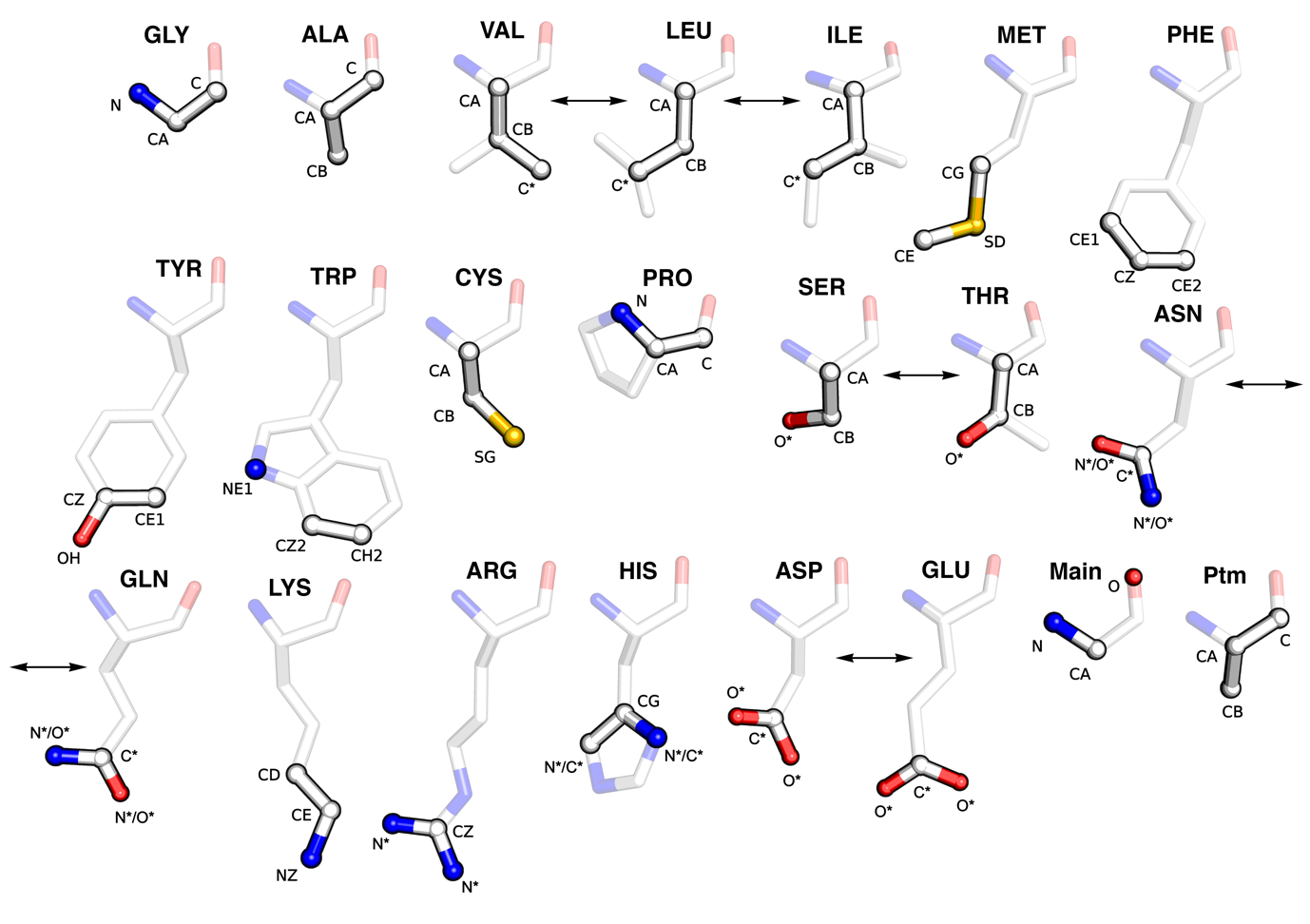


Fig. S1: Functional atoms used in superposition of homologous active sites. For each residue type, three residue-specific atoms are selected (shown in opaque sticks) if the residue functions via its side chain. In cases where a residue functions via its main chain or undergoes post-translational modifications, explicit atom selections are defined, as shown in the lower right part of the figure. Bidirectional arrows indicate residues of equivalent properties that can be superposed interchangeably. Similarly, atoms of symmetrical chemical groups or atoms that are shared between equivalent residues are indicated with a * symbol. These atom selections are a modified version of the definitions from Wallace et al^1^. Models were generated in PyMol^2^.


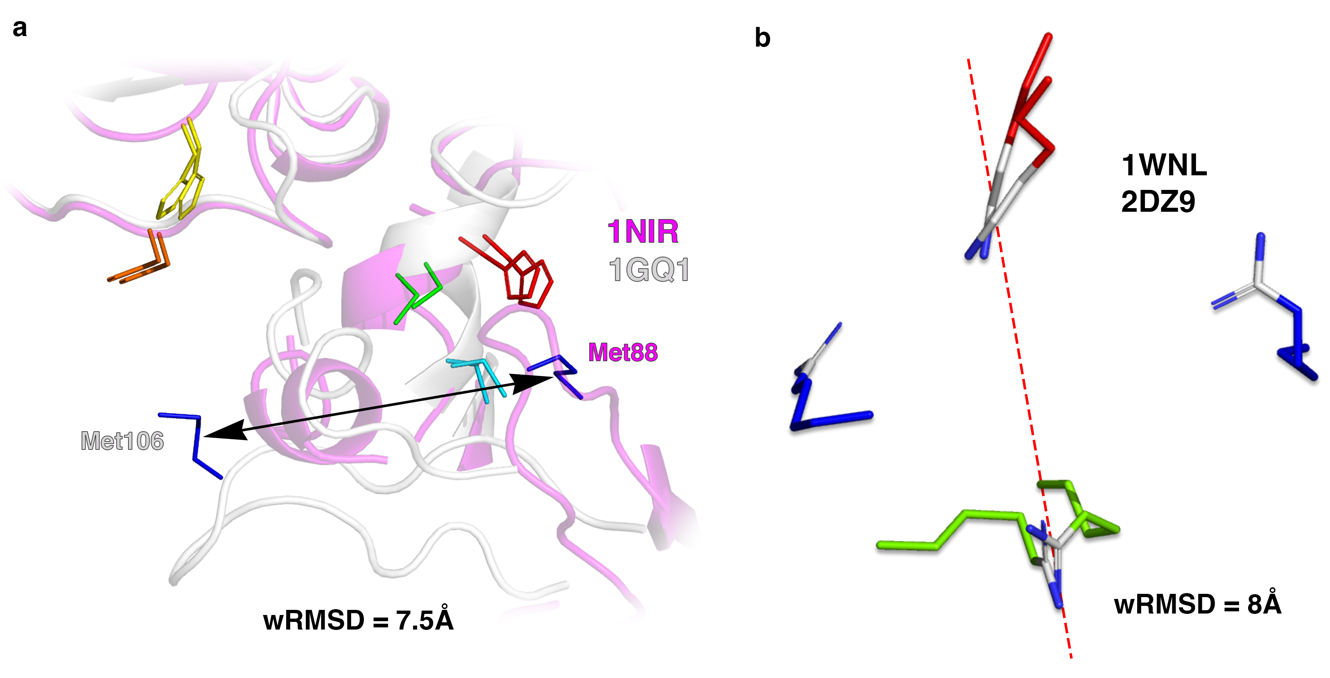


Fig. S2: Outliers in active site structural comparison. a: Two active sites from M-CSA entry 903 (Nitrite reductase, EC 1.7.2.1, PDB IDs: **1NIR**, **1GQ1**) in superposition, showing the unusually high RMSD due to the movement of the loop carrying a methionine residue (blue sticks). Structural transition of the loop is shown with a bidirectional arrow. Catalytic residues are shown as sticks, with equivalent residues from the active site pair shown in the same colour. Met residues on the moving loop are labelled (they are equivalent in the two structures despite the different numbering). Structural context is shown as cartoon. b: Erroneous superposition of two active sites from M-CSA entry 538 (biotin ligase, EC 6.3.4.15, PDB IDs: **1WNL**, **2DZ9**) consisting of three catalytic residues. Due to the geometry of two out of three residues’ functional atoms (they align approximately on the same axis, shown as dashed line), the blue Arg residues are weighted down during fitting iterations, and the two remaining residues (green and red) are free to be superimposed ambiguously. 3D models were prepared in PyMol^2^.

##
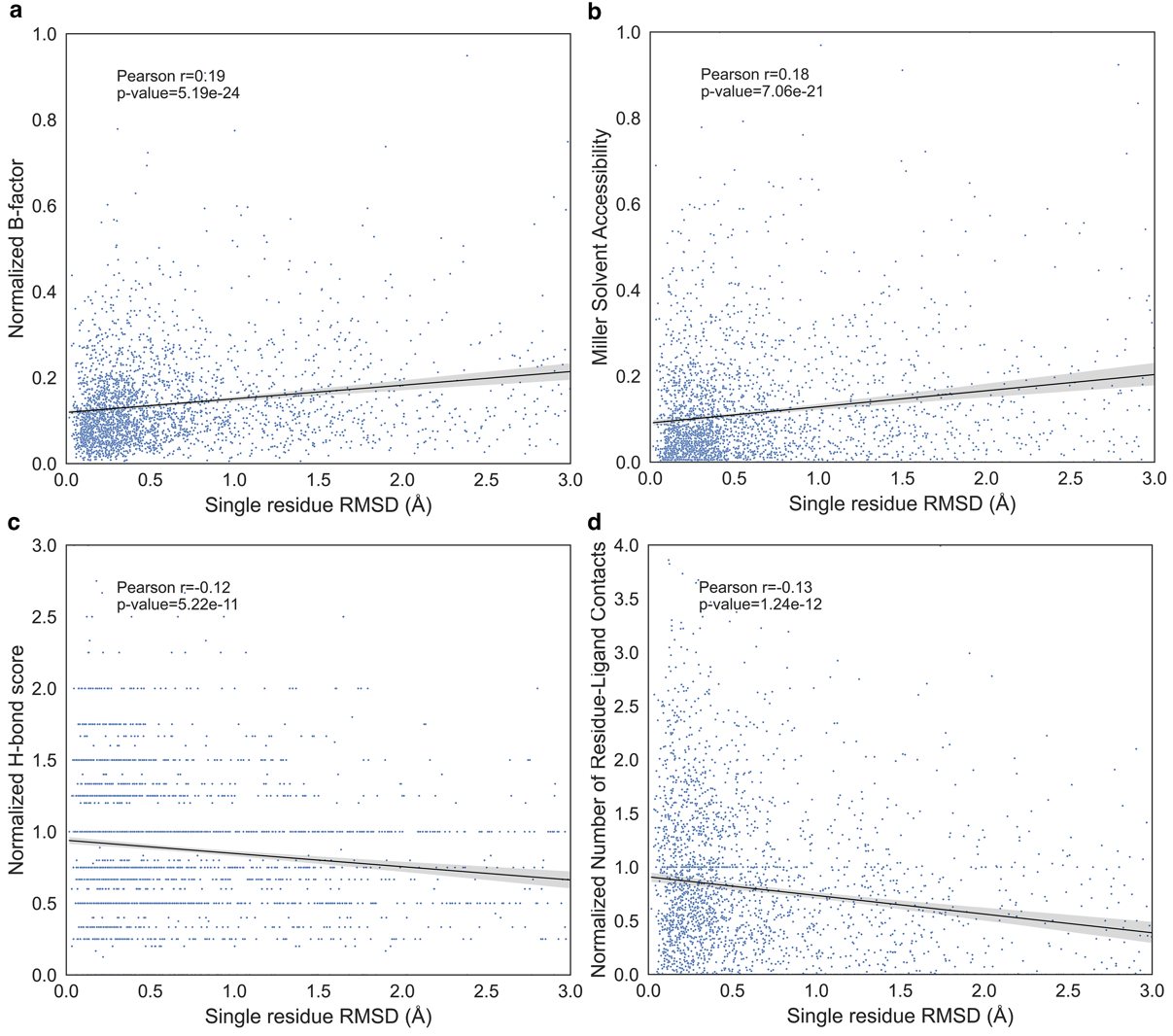


Fig. S3: Correlation between single-residue RMSD versus various structural parameters. a: Normalized B-factor (normalization performed over the Z-score of all B-factors of the structure), b: Miller Solvent Accessibility, c: Normalized H-bond score (expressed as the number of putative polar contacts of donor/acceptor atoms of a residue with neighbouring molecules using a distance threshold of 3.5Å, normalized over the total number of its donor/acceptor atoms), d: Normalized number of residue-ligand contacts (expressed as the number of contacts between atoms of a residue and neighbouring ligands, using a distance threshold of 8Å, normalized of the total number of residue/ligand atoms). Results derive from superposition of active sites within homologous M-CSA families with their respective reference active site. Linear regression was applied in each case, indicated as a line, while the corresponding Pearson correlation coefficients (r) and their p-values are annotated.


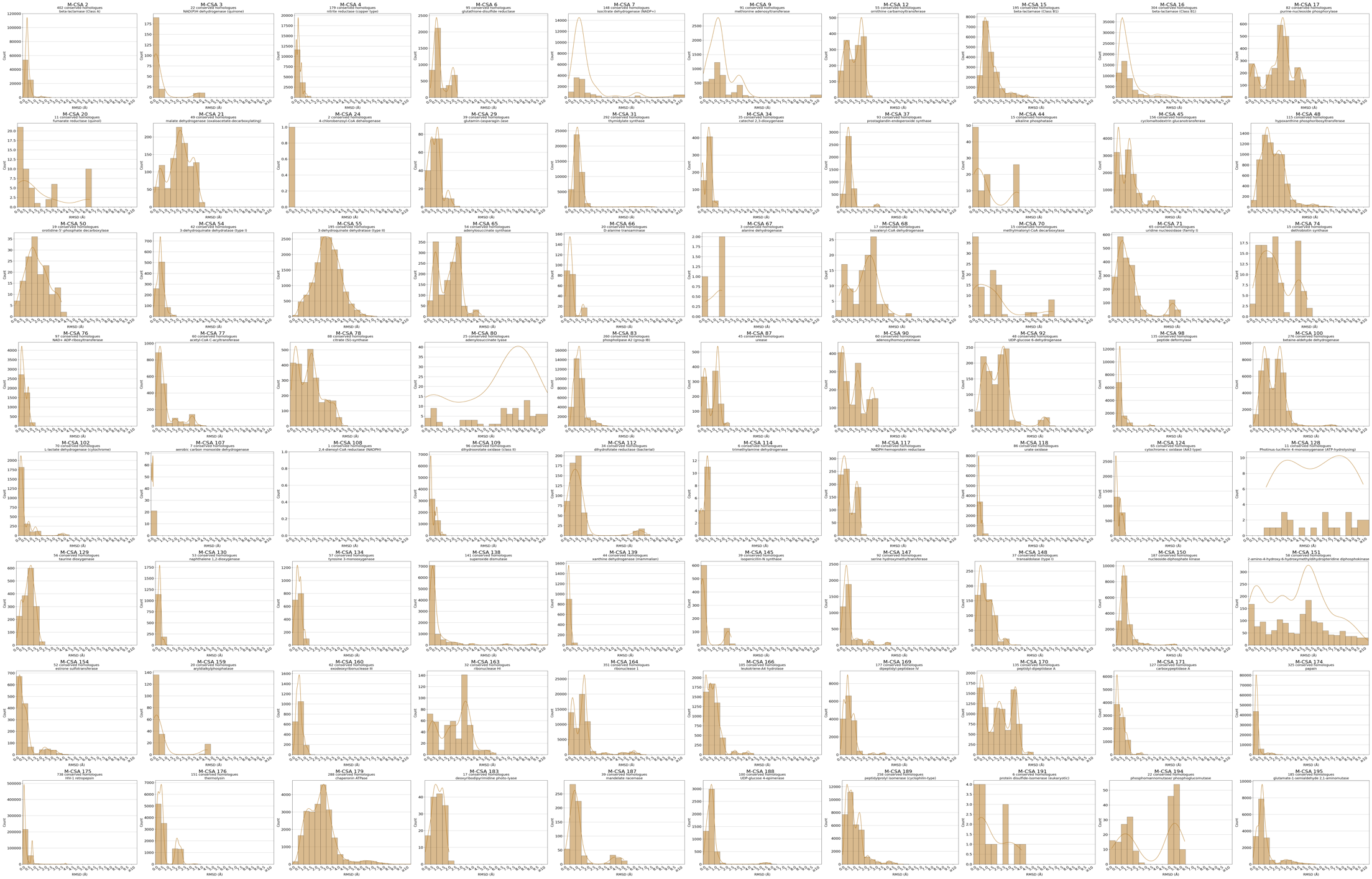

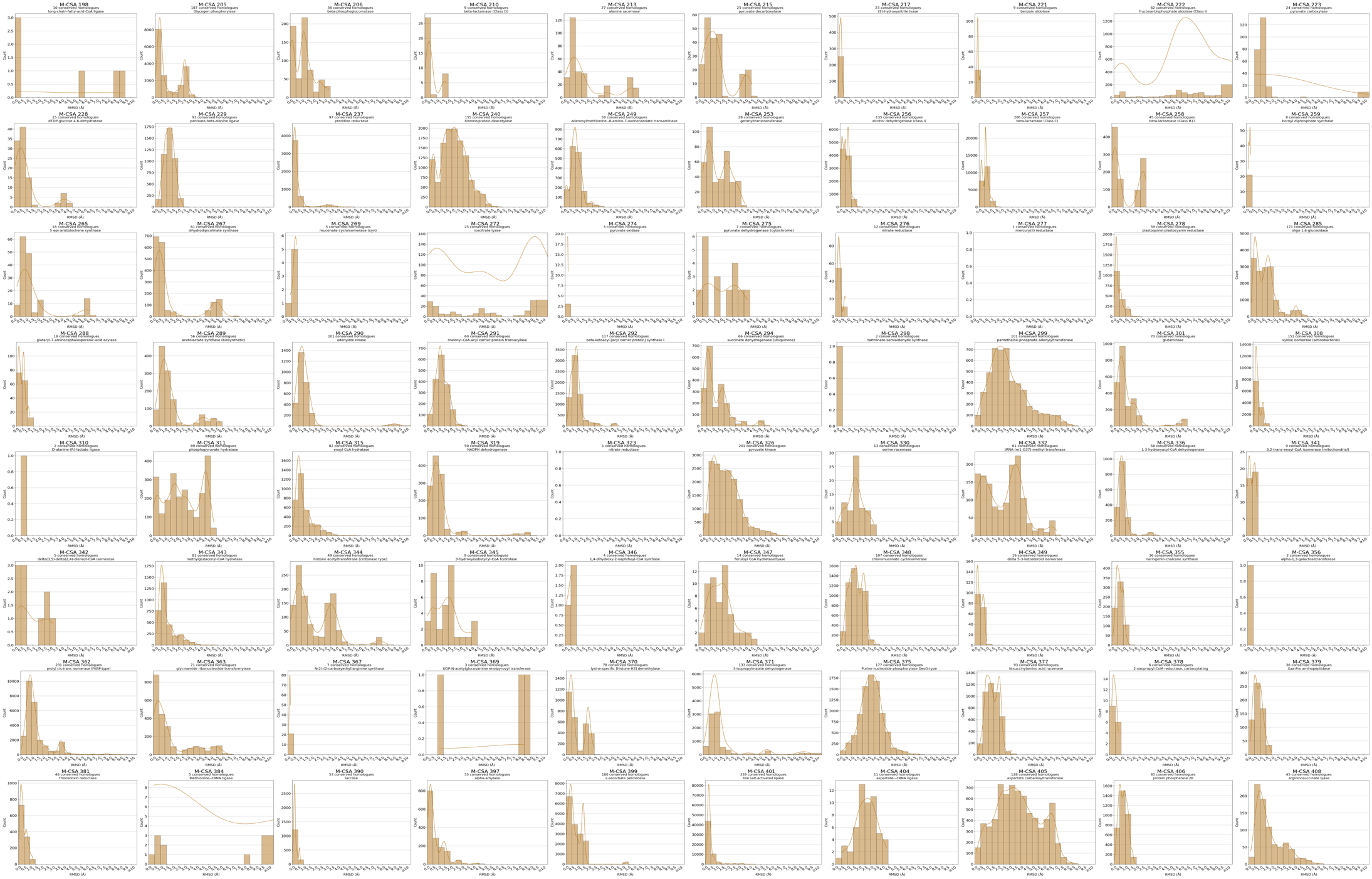

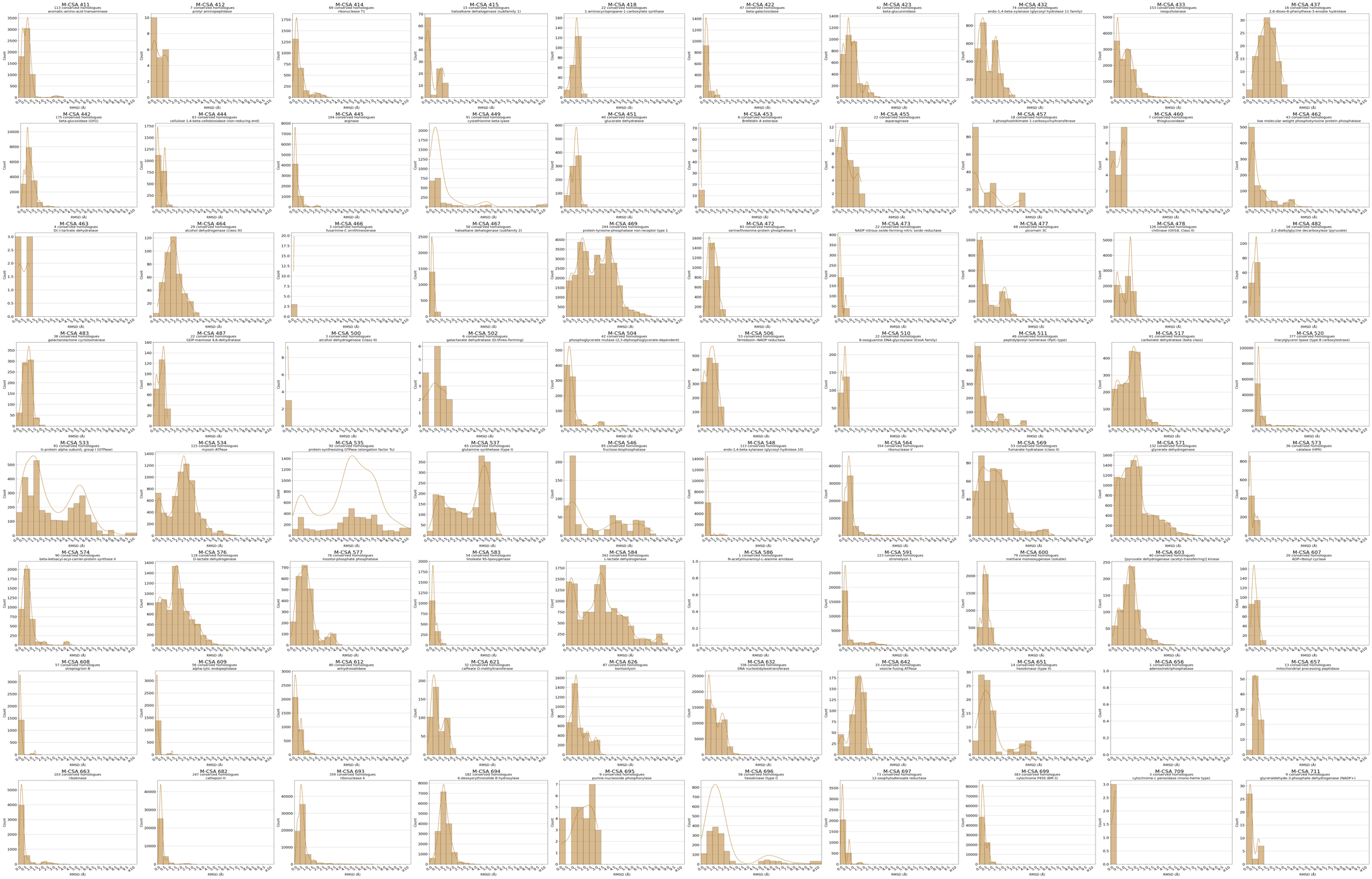

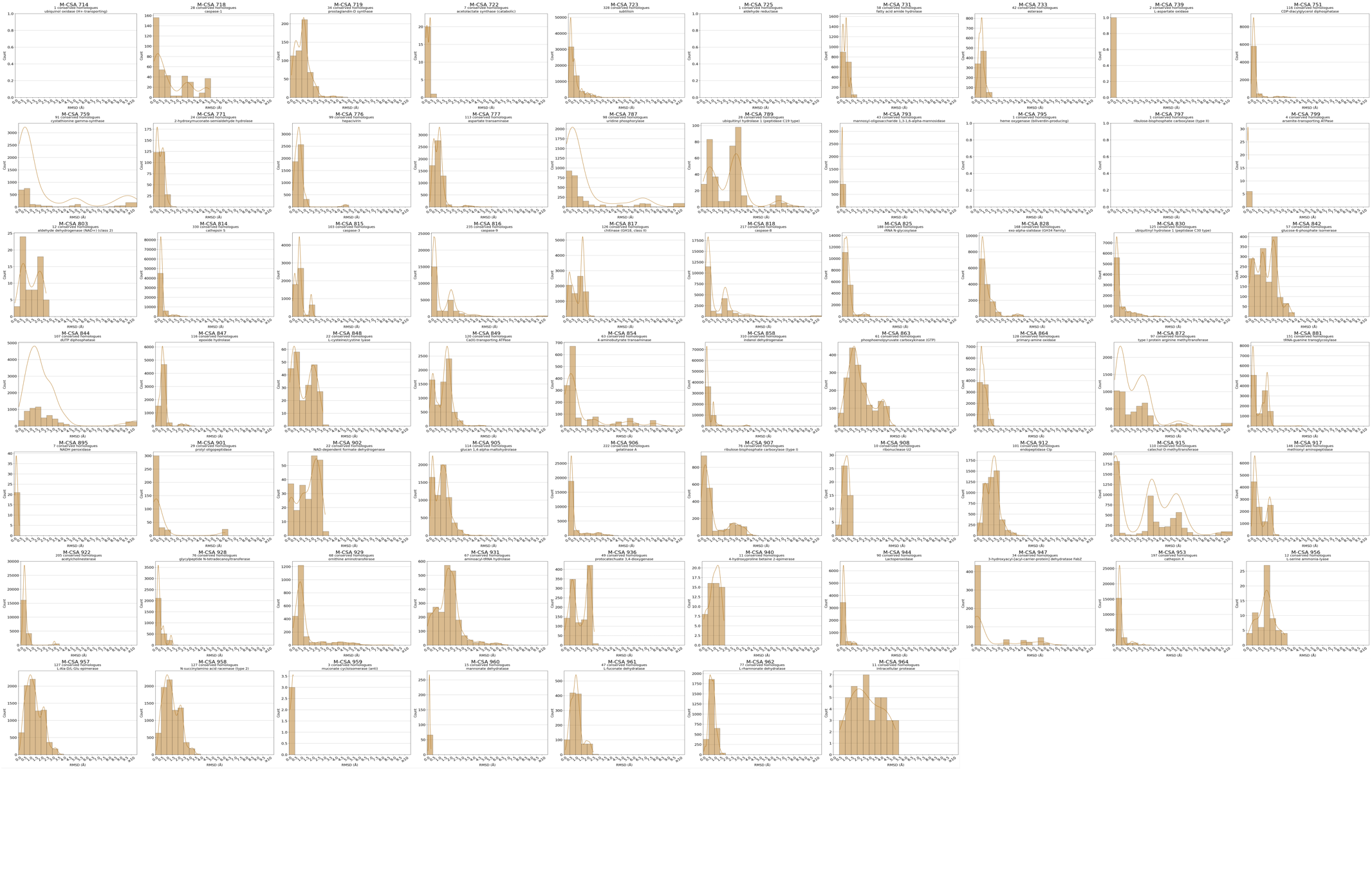


Fig. S4: All-vs-All functional atoms RMSD distributions of conserved active sites, for all M-CSA entries containing at least 50 homologous active site structures. M-CSA ID, enzyme name and number of conserved active sites are annotated on each histogram plot. RMSD scale (x-axis) has a common range for all distributions. RMSD values of ≥10Å are binned altogether for better visualization. Bar width indicate bin width and Kernel density estimation (KDE) is shown as an overlayed curve.


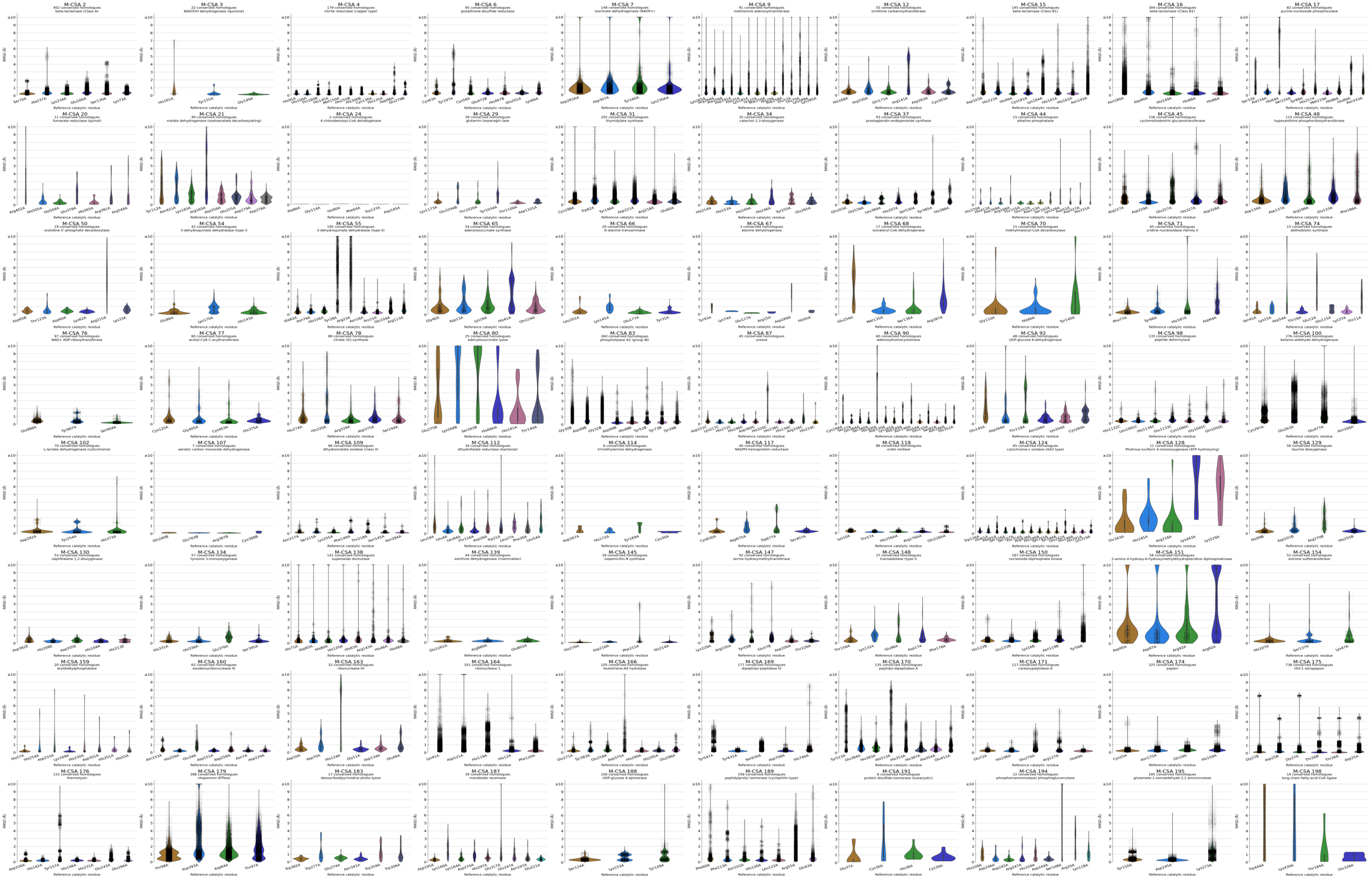

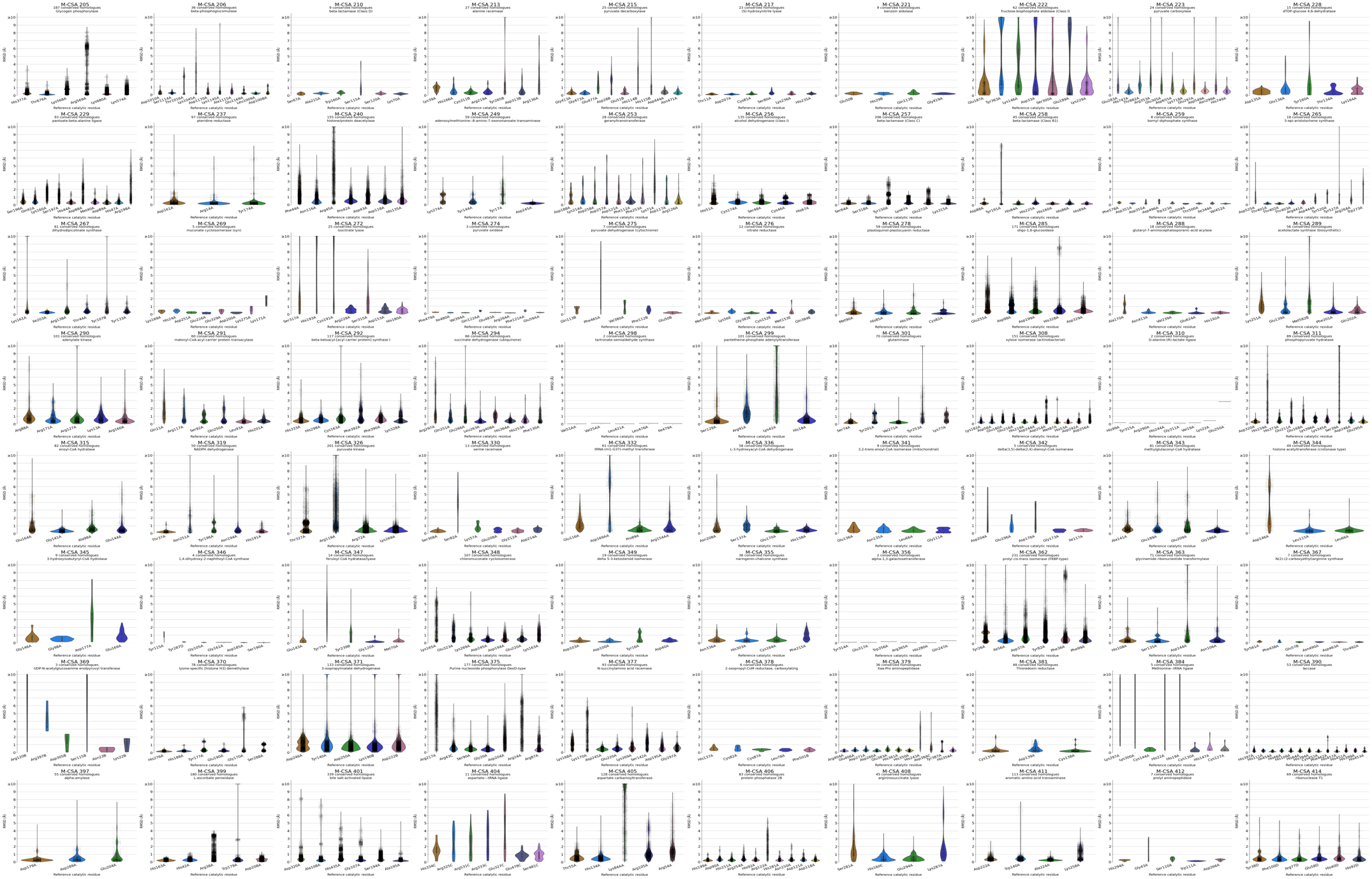

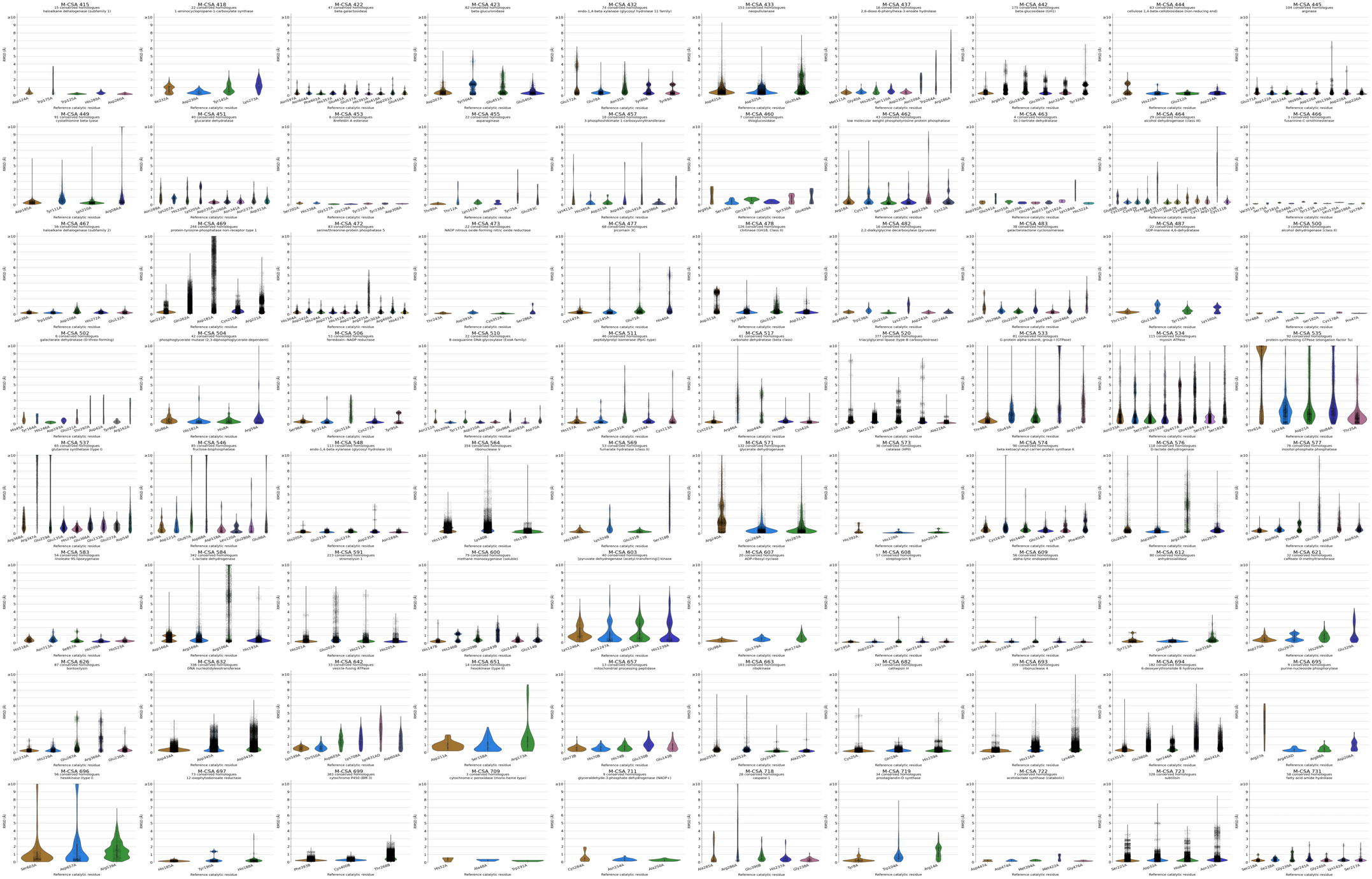

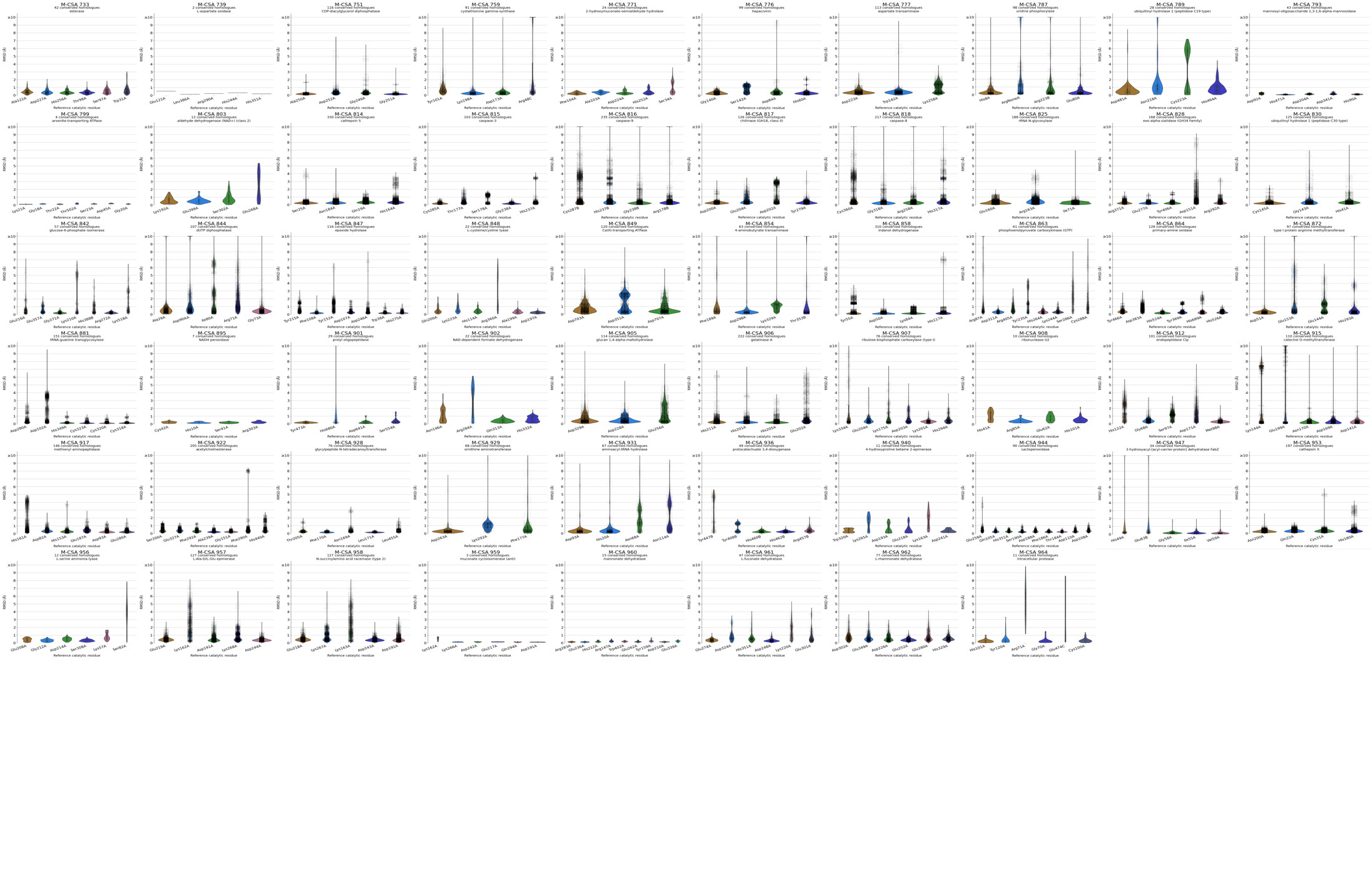


Fig. S5: By-residue functional atoms RMSD distributions (represented as violin plots) of conserved active sites, for all M-CSA entries containing at least 50 homologous active site structures. M-CSA ID, enzyme name and number of conserved active sites are annotated on each plot. Reference residue name, ID and chain ID are annotated on the x-axis. RMSD scale (y-axis) has a common range for all distributions. RMSD values of ≥10Å are binned altogether for better visualization. Strip plots show data point densities and are overlayed to the violin plots.

## References

1. Wallace, A. C., Borkakoti, N. & Thornton, J. M. Tess: A geometric hashing algorithm for deriving 3D coordinate templates for searching structural databases. Application to enzyme active sites. *Protein Science* **6**, 2308–2323 (1997).

2. DeLano, W. L. The PyMOL Molecular Graphics System, Version 2.3. *Schrödinger LLC* (2020).
